# Whole‐Body Magnetic Resonance Imaging in Camurati–Engelmann Disease

**DOI:** 10.1002/jbm4.10357

**Published:** 2020-03-26

**Authors:** Diogo Goulart Corrêa, Clarissa Canella, Alexandra Prufer de Queiroz Campos Araújo, Silvana Mendonça, Flavia Martins Costa

**Affiliations:** ^1^ Department of Radiology, Clínica de Diagnóstico por Imagem (CDPI)/DASA Rio de Janeiro Brazil; ^2^ Department of Radiology Hospital Universitário Antônio Pedro, Federal Fluminense University Niterói Brazil; ^3^ Department of Neuropediatrics Federal University of Rio de Janeiro Rio de Janeiro Brazil

**To The Editor**

We commend Hughes and colleagues^1^ for their study entitled “Observations on the Natural History of Camurati–Engelmann Disease”, published in the *Journal of Bone and Mineral Research*. In this study, 10 members of a family carrying a common *TGFB1* gene mutation had their symptoms evaluated and both skeletal radiographs and scintigraphies performed. Here, we propose a potential role for whole‐body magnetic resonance imaging (WB‐MRI) in evaluations of Camurati–Engelmann disease.

A 7‐year‐old female child presented with chronic fatigue, muscle weakness, and hypotonia, especially in the shoulder and pelvic girdle. Her blood count and serum levels of calcium, potassium, sodium, phosphate, and creatine kinase were normal. A 3‐Tesla WB‐MRI was performed, without sedation, to address a suspicion of inflammatory myopathy. Symmetrical edema was observed in the bone marrow of the diaphyses and metadiaphyseal regions of the femur, tibia, radius, ulna, and humerus. This edema was associated with cortical concentric thickening and irregularities, with bilateral diaphyseal enlargement. Periosteal soft tissue edema was also noted in these regions. No edema was observed in the epiphyseal regions (Fig. 1). The scan took around 35 min. A genetic analysis revealed a heterozygous mutation present in the patient's *TGFB1* gene. None of the child's family members presented similar symptoms.

**Figure 1:**
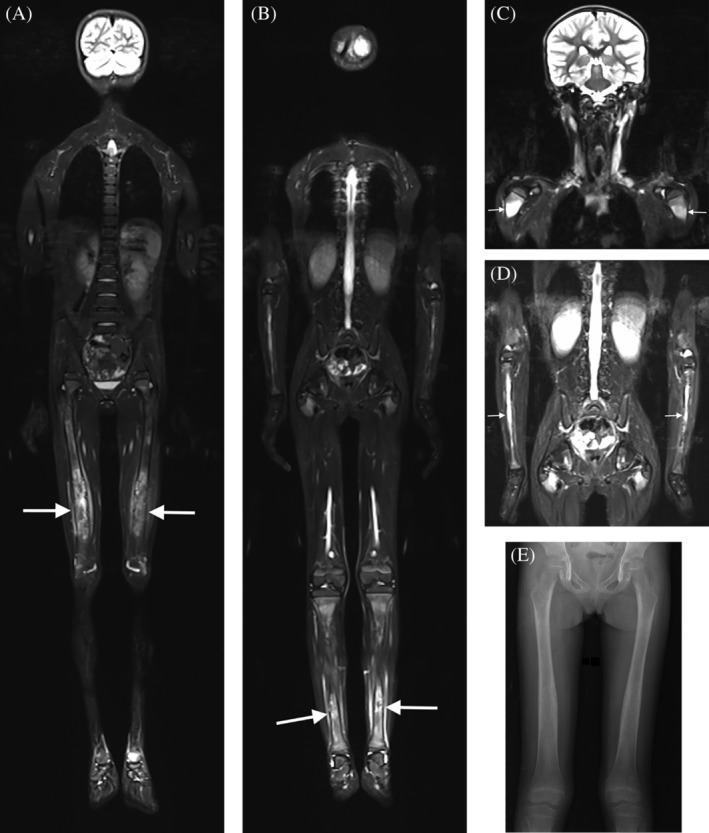
Whole‐body magnetic resonance imaging in Camurati–Engelmann disease. Whole‐body short tau inversion recovery in the coronal plane shows symmetrical edema in the bone marrow of the diaphyses and metadiaphyseal regions of the bilateral femur (arrows in *A*) and tibia (arrows in *B*). Segmental images of the superior parts of the body show edema in the bone marrow of the diaphyses and metadiaphyseal regions of the humerus (arrows in *C*) and radius (arrows in *D*). Cortical concentric thickening and irregularities, with diaphyseal enlargement, were also observed. Note that the epiphyseal regions are spared. There was no restricted diffusion. (*E*) A femoral radiograph shows only slight fusiform cortical thickening of the femoral diaphysis.

Over the past few years, WB‐MRI has gained greater attention with a rapidly growing number of indications. WB‐MRI allows lesions to be detected and comprehensive evaluations of treatment response to be conducted in many oncologic and nononcologic disorders. In addition, WB‐MRI has a clinically viable duration and excellent soft tissue contrast, especially when performed with 3‐Tesla scanners.^2^ WB‐MRI can cover either the body from head to toe or it can be more limited (eg, from “eyes to thighs”), depending on the basis of the examination. Overall, WB‐MRI provides exquisite anatomical resolution with excellent tissue contrast and high sensitivity to bone and soft tissue alterations.^2^, ^3^

Initially, WB‐MRI was used to assess the entire skeleton in oncology applications. Subsequently, WB‐MRI has emerged as a technique for detecting and quantifying bone metastasis, multiple myeloma, and lymphoma. For these indications, WB‐MRI has been found to outperform bone scintigraphy and radiographs for lesion detection.^3^ Thus, it has become the modality of choice for disease staging and treatment monitoring.

In addition to oncologic applications, WB‐MRI is progressively being applied to the detection and characterization of benign multisystemic and multifocal diseases that affect bones, joints, tendons, entheses, or neurovascular structures.^3^ For example, the main benign diseases that are assessed by WB‐MRI include seronegative spondyloarthropathies, synovitis, acne, pustulosis, hyperostosis, osteitis (SAPHO) syndrome, systemic sclerosis, and idiopathic inflammatory myopathies.

In pediatric patients, WB‐MRI has the advantage of providing a comprehensive evaluation of the body without exposure to ionizing radiation. WB‐MRI also reduces the number of examinations performed under sedation. Currently, WB‐MRI is prescribed in pediatric cases of chronic recurrent multifocal osteomyelitis (CRMO), juvenile idiopathic arthritis, and juvenile dermatomyositis, as well as for pediatric patients with fevers of unknown origin.^4^

In Camurati–Engelmann disease, MRI can show exuberant diffuse cortical thickening in long bones diaphysis in addition to increases in diaphysis diameter. In fluid‐sensitive sequences, such as T2‐weighted imaging with fat saturation and short tau inversion recovery (STIR), intramedullary hyperintense signals are observed. Contrast enhancement after i.v. gadolinium injection is also observed due to edema and inflammatory activity, secondary to increased bone turnover. Furthermore, MRI can detect muscle atrophy. MRI is also as effective as CT for demonstrating hyperostotic bone and compressive effects on cranial nerves.^5^

To the best of our knowledge, the use of WB‐MRI in cases of Camurati–Engelmann disease has not been evaluated. However, previous reports have described the use of WB‐MRI in other skeletal dysplasias such as Trevor disease. WB‐MRI can identify unsuspected lesions outside the limited field of view of the usual MRI scan or conventional radiography, and can identify characteristic distribution patterns of lesions. This ability to detect additional unknown lesions represents a clear therapeutic impact of WB‐MRI.^6^

The clinical applicability of WB‐MRI has contributed to greater recognition and use of this technique as a state‐of‐the‐art evaluation method for various diseases. However, further investigation is needed to develop the technique for more clinical purposes and to reduce its cost, in addition to evaluate the advantages of WB‐MRI over standard radiographic studies for early lesion detection, for evaluation of lesions distribution, and for treatment evaluation in bone dysplasias such as Camurati–Engelmann disease.

## Disclosure

The authors have no conflicts of interest to declare.
